# Statistical Issues: Barr et al. Respond

**Published:** 2006-12

**Authors:** Dana B. Barr, Doug Landsittel, Marcia Nishioka, Kent Thomas, Brian Curwin, James Raymer, Kirby C. Donnelly, Linda McCauley, P. Barry Ryan

**Affiliations:** National Center for Environmental Health, Centers for Disease Control and Prevention, Atlanta, Georgia, E-mail: dbarr@cdc.gov; Department of Mathematics and Computer Science, Duquesne University, Pittsburgh, Pennsylvania; Battelle Memorial Institute, Columbus, Ohio; National Exposure Research Laboratory, Office of Research and Development, U.S. Environmental Protection Agency, Research Triangle Park, North Carolina; National Institute for Occupational Safety and Health, Centers for Disease Control and Prevention, Cincinnati, Ohio; RTI International, Research Triangle Park, North Carolina; Texas A&M University System, Health Science Center, College Station, Texas; School of Human Environmental Sciences, University of Pennsylvania, Philadelphia, Pennsylvania; Rollins School of Public Health, Emory University, Atlanta, Georgia

Mage et al. criticize our article (Barr et al. 2006), stressing six “… factual and conceptual errors that need to be called to the readers’ attention.” We appreciate their careful reading of our work, and they raise several important points regarding survey design. However, we take issue with some of their statements.

Many investigations are designed to generalize the results of the research performed within a sample population to a larger population. In these types of investigations, enrollment of a representative sample is a necessary condition for making inferences to the larger population through known selection probabilities that are then used for applying sampling weights to study results. However, in order to generalize the results, these studies must have an adequate sample size, high response rate, and, importantly, a preliminary assessment of whether the factors for probability selection and weighting will be relevant to the condition being measured. Although representative samples are desirable and have been achieved in many studies, some studies in rare or difficult-to-reach populations cannot practically meet the criteria mentioned above.

Farmworkers are often not the ones applying pesticides (i.e., they are not applicators); quite often these farmworkers are unaware of the actual pesticides being applied or when they are applied. The potential for undue exposure may be more likely if farmworkers are not properly informed of the application or reentry times or if they do not understand the potential exposure scenarios.

Research investigations involving farmworker exposures can present particular difficulties in selecting a representative sample of the population. Data for developing relevant sampling frames and selection probabilities are often limited by demographic and work factors. Obtaining high response rates for farmworkers can also be challenging because of problems associated with access, high mobility, geographic dispersion, trust, and cultural practices (Arcury et al. 2006). However, these populations remain important and potentially vulnerable populations that should be included in research investigations, even if the conditions for using a probability sample cannot always practically be met.

In any particular situation, the decision to use probability sampling will depend on the hypothesis. However, important and relevant research questions can be investigated without selecting a representative sample of the population, as noted by Mage et al. regarding particle exposure studies in high-risk subpopulations. Nonprobability samples can provide useful information on particular hard-to-reach populations, for intensive examination of conditions and factors related to exposures, or for hypothesis generation. By forcing all studies to conform to the same design, we may not be able to answer specific research questions.

The studies cited by Mage et al. were designed as probability samples, but each had differential drop-out rates during selection and sampling. Potential drop-out non-representativeness can be accounted for if the effect on exposure or the outcome variable is known. However, in some populations, the details of the accounting are not easily accomplished. Mage et al. cited the National Health and Nutrition Examination Survey (NHANES) as “another excellent example of proper probability-based sample selection.” However, even NHANES III (1988–1994) used a nonprobability sample for the environmental subset (Hill et al. 1995). These data provided an invaluable first look at U.S. population exposures and served as a basis to add statistical sampling for environmental chemicals to the current NHANES series. These data have also been used to estimate doses in the U.S. population for comparison to the U.S. Environmental Protection Agency’s reference doses (Mage et al. 2004). In fact, it is often difficult to design a population-based study without preliminary data.

We have participated in the design and implementation of studies using both probability and nonprobability sampling that have added invaluable information on various population exposures. We recognize the practical difficulties and challenges for meeting the criteria for representative sampling in farmworker populations and also the important information that such studies can provide. Although we agree that without a probability sample, the results should only apply to individuals in studies and should not be generalized to a population, we disagree with the contention made by Mage et al. that no useful information can come from studies using samples that do not fulfill their criteria.

## References

[b1-ehp0114-a00689] Arcury TA, Quandt SA, Barr DB, Hoppin JA, McCauley L, Grzywacz JG (2006). Farmworker exposure to pesticides: methodologic issues for the collection of comparable data. Environ Health Perspect.

[b2-ehp0114-a00689] Barr DB, Landsittel D, Nishioka M, Thomas K, Curwin B, Raymer J (2006). A survey of laboratory and statistical issues related to farmworker exposure studies. Environ Health Perspect.

[b3-ehp0114-a00689] Hill RH, Head SL, Baker S, Gregg M, Shealy DB, Bailey SL (1995). Pesticide residues in urine of adults living in the United States: reference range concentrations. Environ Res.

[b4-ehp0114-a00689] Mage DT, Allen RH, Gondy G, Smith W, Barr DB, Needham LL (2004). Estimating pesticide dose from urinary pesticide concentration data by creatinine correction in the Third National Health and Nutrition Examination Survey (NHANES-III). J Expo Anal Environ Epidemiol.

